# High‐Performance Ammonium Cobalt Phosphate Nanosheet Electrocatalyst for Alkaline Saline Water Oxidation

**DOI:** 10.1002/advs.202100498

**Published:** 2021-05-16

**Authors:** Zhongxin Song, Kaixi (Cathy) Wang, Qian Sun, Lei Zhang, Junjie Li, Dingjiu Li, Pok‐Wai Sze, Yue Liang, Xueliang Sun, Xian‐Zhu Fu, Jing‐Li Luo

**Affiliations:** ^1^ Shenzhen Key Laboratory of Polymer Science and Technology Guangdong Research Center for Interfacial Engineering of Functional Materials College of Materials Science and Engineering Shenzhen University Shenzhen 518060 China; ^2^ Department of Mechanical and Materials Engineering University of Western Ontario London ON N6A 5B9 Canada; ^3^ Department of Electrical and Computer Engineering Waterloo Institute for Nanotechnology University of Waterloo 200 University Avenue West Waterloo ON N2L 3G1 Canada

**Keywords:** alkaline saline water oxidation, ammonium cobalt phosphate, electrocatalysts, nanosheets, oxygen evolution reaction

## Abstract

The development of highly efficient electrocatalysts toward the oxygen evolution reaction is imperative for advancing water splitting technology to generate clean hydrogen energy. Herein, a two dimensional (2D) nanosheet ammonium cobalt phosphate hydrate (NH_4_CoPO_4_·H_2_O) catalyst based on the earth‐abundant non‐noble metal is reported. When used for the challenging alkaline saline water electrolysis, the NH_4_CoPO_4_·H_2_O catalyst with the optimal thickness of 30 nm achieves current densities of 10 and 100 mA cm^−2^ at the record low overpotentials of 252 and 268 mV, respectively, while maintaining remarkable stability during the alkaline saline water oxidation at room temperature. X‐ray absorption fine spectra reveal that the activation of Co (II) ions (in NH_4_CoPO_4_·H_2_O) to Co (III) species constructs the electrocatalytic active sites. The 2D nanosheet morphology of NH_4_CoPO_4_·H_2_O provides a larger active surface area and more surface‐exposed active sites, which enable the nanosheet catalyst to facilitate the alkaline freshwater and simulated seawater oxidation with excellent activity. The facile and environmentally‐benign H_2_O‐mediated synthesis route under mild condition makes NH_4_CoPO_4_·H_2_O catalyst highly feasible for practical manufacturing. In comparison with noble metals, this novel electrocatalyst offers a cost‐effective alternative for economic saline water oxidation to advance water electrolysis technology.

## Graphical abstract

A two dimensional structured ammonium cobalt phosphate nanosheet is fabricated by a low‐energy consuming H_2_O‐mediated synthesis route at room temperature. Through adjusting the thickness of nanosheets, the optimal catalyst with high surface area and exposed active sites is achieved, which exhibits high electrocatalytic activity and stability for the oxygen evolution reaction in alkaline saline water electrolyte.

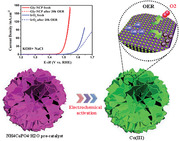

## 1 Introduction

With the increasing global warming and environmental pollution negatively affecting our living habitat, seeking alternative clean energy in replacement of fossil fuels has become an imperative task and attracted great attentions globally. Hydrogen (H_2_) as the carbon‐neutral energy carrier is considered as one of the most promising clean and renewable energy sources.^[^ ^1^ ^]^ For sustainable H_2_ production, electrocatalytic water splitting presents itself as a promising future technology.^[^ ^2^ ^]^ Generally, overall water splitting by electrolysis consists of cathodic hydrogen evolution reaction (HER) and anodic oxygen evolution reaction (OER).^[^ ^3^ ^]^ The bottleneck of water splitting is anodic OER due to the sluggish reaction kinetics which requires high overpotential for delivering high current density.^[^ ^4^ ^]^ The state‐of‐the‐art OER electrocatalysts are highly depend on noble metal‐based catalysts such as RuO_2_ and IrO_2_, especially in acidic conditions.^[^ ^5^ ^]^ However, the large‐scale application of the noble metal

electrocatalysts is greatly hindered by their high cost and poor stability in driving the OER under acidic electrolyte. To reduce the cost, it is excited to note that some non‐noble‐metal‐based electrocatalysts are able to catalyze OER with remarkable catalytic activity. However, it is a tough challenge for non‐noble metal electrocatalysts to work stably in acidic media due to the metal dissolution issue. This serves as a strong driving force to stimulate the investigations of non‐noble‐metal electrocatalysts in alkaline conditions. Developing the low‐cost and high‐performance OER catalyst which exhibits high stability and corrosion resistance is one of the significant challenges for water splitting in alkaline media. Moreover, for mass production of H_2_ fuel, use of seawater instead of freshwater can be highly advantageous because of the enormous natural resource of seawater on the surface of our planet.^[^ ^6^ ^]^ However, the aggressive anions originate from seawater would cause electrode corrosion during the anodic reaction, which is one of the tough issues in seawater oxidation.^[^ ^7^ ^]^ To tackle these challenges, the economic electrocatalysts based on earth‐abundant non‐noble metals with highly active and stable OER performance need to be developed, especially in alkaline seawater electrolysis.

Transition metals and their derivatives are attractive OER catalyst candidates owing to their activity and potential stability during seawater electrolysis.^[^ ^8^ ^]^ Very recently, a few pioneering studies have described the successful generation of transition metal phosphides toward water oxidation with encouraging results.^[^ ^9^ ^]^ For example, Li and co‐workers reported the use of N‐doped carbon nanotubes (NCNTs) decorated with CoP nanoparticles as the electrocatalyst for the OER, which exhibits an overpotential of 310 mV to achieve the current density of 10 mA cm^−2^ in 1.0 m KOH.^[^ ^9a^ ^]^ Driess and co‐workers presented a crystalline lazulite‐like cobalt phosphate, Co_3_(OH)_2_(HPO_4_)_2_, as an efficient precatalyst for the OER in alkaline media. In a standard three‐electrode system, the Co_3_(OH)_2_(HPO_4_)_2_ catalyst displayed an overpotential of 240 mV for OER to deliver 10 mA cm^−2^, which is lower than that of the state‐of‐the‐art RuO_2_ and IrO_2_ catalysts.^[^ ^9b^ ^]^ Moreover, transition metal hydroxides, such as NiFe hydroxide,^[^ ^8d^ ^]^ S‐doped Ni/Fe (oxy)hydroxide,^[^ ^10^ ^]^ Ni/NiS*_x_*/NiFe hydroxide,^[^ ^8f^ ^]^ bimetallic phosphide (Ni_2_P‐Fe_2_P) ^[^ ^11^ ^]^ and sodium cobalt‐iron pyrophosphate^[^ ^7c^ ^]^ have been investigated as OER electrocatalysts under alkaline simulated seawater conditions. Although tremendous research efforts have been devoted to developing efficient transition metal phosphate electrocatalysts for water electrolysis under freshwater conditions, the performance in seawater electrolysis remains quite limited. Additionally, the synthesis processes for these catalysts are usually timely tedious and of high energy consumption, which may restrict their practical applications. Therefore, it necessitates the need to develop cost‐effective transition metal phosphate catalysts using facile synthesis methods for high‐performance seawater electrolysis.

Herein, we report the synthesis of the two dimensional (2D) ammonium cobalt phosphate hydrate (NH_4_CoPO_4_·H_2_O) nanosheet, referred to as NCP, as a high performance electrocatalyst for the OER in alkaline simulated seawater electrolyte. The NCP nanosheets are prepared by a facile and environmentally‐benign H_2_O‐mediated synthesis route at room temperature, this simplicity makes NCP catalyst highly favorable in terms of practical industrial manufacturing. The rational design of transition‐metal phosphate with 2D nanosheet structure^[^ ^12^ ^]^ has been proposed to be beneficial in providing large surface area, therefore, the high density of surface‐exposed active sites. The non‐noble metal of Co‐based compounds has been reported to show good OER activity. Among the Co derivatives, Co‐based phosphate show superior intrinsic stability in the alkaline electrolyte, therefore 2D NCP nanosheet was chosen as the anode materials for the OER. Working as the OER catalyst, the NCP nanosheets with the thickness of 30 nm exhibit low overpotentials of merely 265 and 252 mV to deliver 10 mA cm^−2^ current density in alkaline water (1.0 m KOH) and simulated seawater (1.0 m KOH + 0.5 m NaCl), respectively, significantly lower than those of IrO_2_ catalyst counterpart. Furthermore, the optimal thin NCP catalyst exhibits highly durable electrochemical stability during continuous 20 h OER under the challenging alkaline saline water condition. Notably, this is the first report on the application of NCP nanosheet as an electrocatalyst toward saline water oxidation in light of the nature of this challenging environment. Our results suggest that NCP nanosheet is a promising catalyst candidate for OER under both alkaline freshwater and saline water media, which can contribute to the scientific and technological development of seawater electrolysis.

## 2 Results and Discussion

### 2.1 Synthesis and Characterization of NCP Catalysts

**Figure 1:**
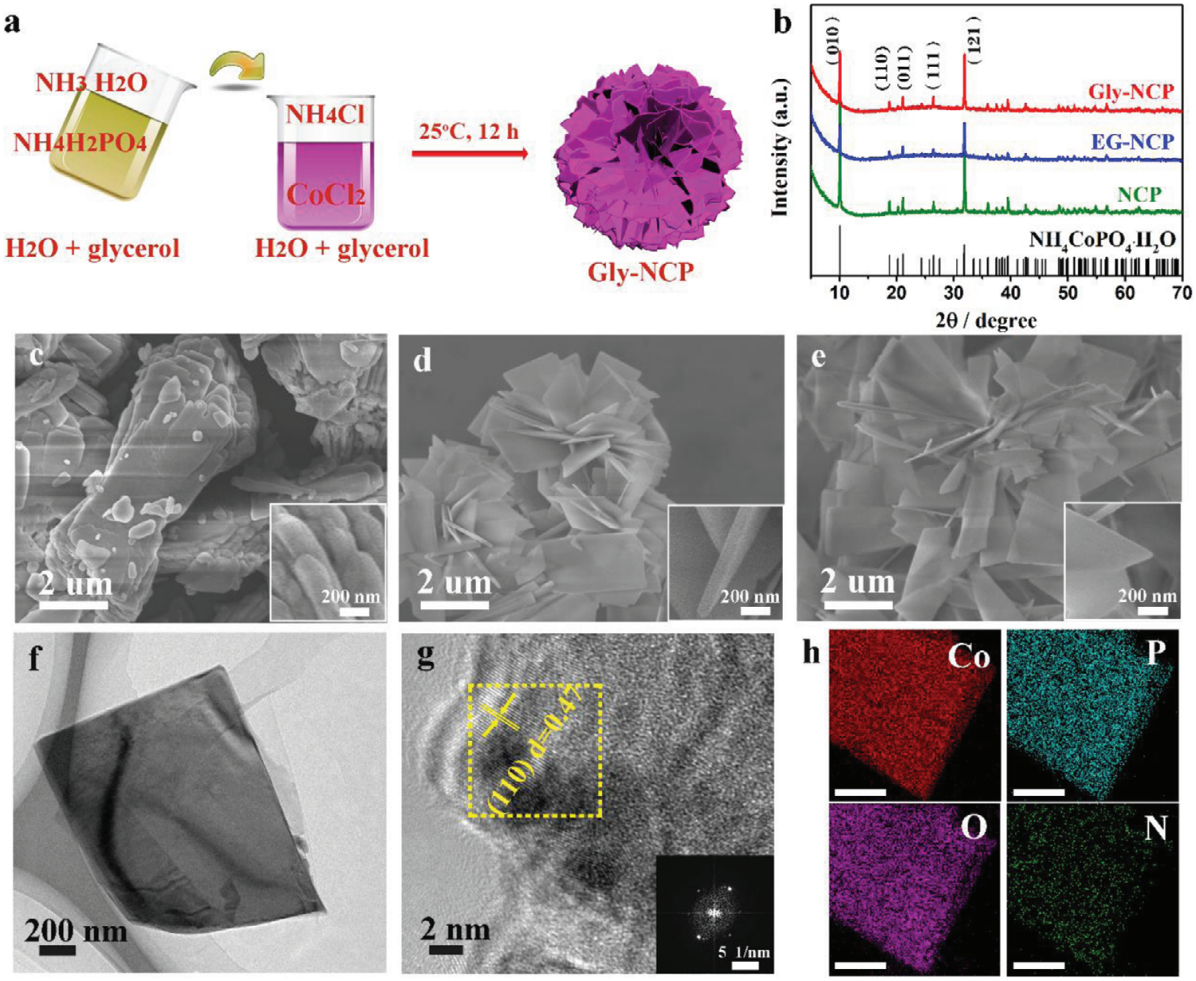
Synthesis and microscopic characterization of the as‐prepared NCP, EG‐NCP, and Gly‐NCP materials. a) Schematic illustration of the synthesis procedure for Gly‐NCP nanosheets. b) XRD patterns of the as‐prepared NCP samples. The SEM images of c) NCP, d) EG‐NCP, and e) Gly‐NCP at two magnifications. f) TEM image, g) HRTEM image, and SAED pattern (inset) obtained from the yellow rectangular region, and h) EDS elemental maps of one Gly‐NCP nanosheet.

In this study, CoCl_2_ and NH_4_H_2_PO_4_ were respectively utilized as metal and phosphate precursors for the synthesis of NCP nanosheets via one‐pot chemical precipitation technique. The preparation of NCP with controlled thickness is schematically illustrated in **Figure** **1a**, and the obtained samples are named as NCP, EG‐NCP, and Gly‐NCP which are prepared under the solvent of water, water+ethylene glycol (EG), and water+glycerol (Gly), respectively. The phase purity and crystallographic structure of the as‐prepared NCP, EG‐NCP, and Gly‐NCP samples were studied by X‐ray diffraction (XRD). XRD patterns in Figure 1b indicate that the as‐prepared NCP materials with crystalline morphology are obtained under synthesis conditions. As shown in Figure 1b, all diffraction peaks of NCP samples can be well indexed to the orthorhombic NH_4_CoPO_4_·H_2_O (JCPDS No.21‐0793). The two dominant peaks of 2*θ* at 10.1° and 31.8° correspond to the (001) and (121) planes of NH_4_CoPO_4_·H_2_O. The sharp diffraction peaks suggest the highly crystalline nature of NCP and that no impurity phases are introduced by the chemical precipitation process at room temperature.

The morphological evolution of NCP with different synthetic solvents was studied using scanning electron microscopy (SEM). Figure 1c shows the SEM image of NCP prepared in deionized (DI) water and a platelike structure of NCP is displayed. The layered arrangement of nanosheets with an average size of 5.0 *μ*m and thickness of 250 nm is observed in the NCP, which shows similar morphological size with the reported literature^[^ ^13^ ^]^ (200–300 nm plate thickness). Figure 1d displays the SEM image of EG‐NCP. Different from platelike NCP, the EG‐NCP is found to have formed 3D flowerlike morphology by connecting the nanosheets with the thickness of 100 nm. Moreover, in the case of H_2_O+glycerol solvent, the as‐obtained Gly‐NCP exhibits a similar flowerlike morphology to that of EG‐NCP_,_ while the nanosheets of Gly‐NCP demonstrate a much thinner thickness of 30 nm (Figure 1e). The SEM results reveal that the morphology and nanosheet thickness of NCP samples can be easily controlled through adjusting the solvent conditions. The difference among platelike NCP, flowerlike EG‐NCP, and Gly‐NCP involves the arrangement and thickness of the nanosheets. In the mixed solution of H_2_O+EG and H_2_O+glycerol, the solvent viscosity value is higher than that of DI water. Due to the Ostwald ripening process in the thick solution, the nanosheets grow randomly and integrate with each other, which results in flower‐like morphology of EG‐NCP and Gly‐NCP. In terms of NCP, the nanosheets are thicker and wider and are not integrated with each other. The transmission electron microscopy (TEM) image in Figure 1f further details the rectangle nanosheet structure of the Gly‐NCP. The high resolution TEM (HRTEM) image and the energy dispersive X‐ray spectroscopy (EDS) mappings are obtained by zoom on the edge area of one nanosheet from Gly‐NCP. Figure 1g reveals the distinctive lattice fringes with interplanar spacing of 0.47 nm, which is assigned to the (110) plane of Gly‐NCP. The selected area electron diffraction (SAED) pattern (inset) is obtained from the yellow rectangular region in Figure 1g. The EDS mappings (Figure 1h) for a Gly‐NCP nanosheet clearly display that Co, P, N, and O are homogeneously distributed throughout the Gly‐NCP. All the above results prove that the low‐energy consuming one‐pot chemical precipitation is an effective approach to fabricating the NCP nanosheets. Moreover, it can be found that the solvent environment also plays a significant role in tuning the nanosheet thickness and morphology.

**Figure 2:**
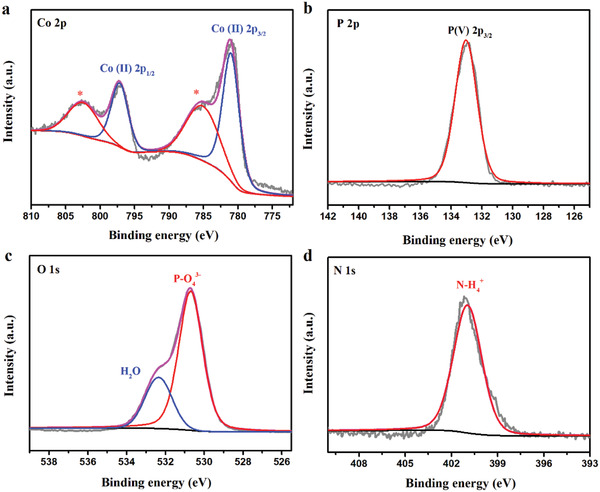
High‐resolution XPS of a) Co 2p, b) P 2p, c) O 1s, and d) N 1 s of the as‐prepared Gly‐NCP.

X‐ray photoelectron spectroscopy (XPS) measurements were performed to analyze the chemical characteristics of the as‐prepared Gly‐NCP sample. The XPS spectra survey in Figure S1 (Supporting Information) shows that Co (11.7 at%), P (14.5 at%), O (62.6 at%), and N (11.2 at%) are the basic elements in constructing Gly‐NCP. The core peaks at 781.0 and 797.2 eV can be associated with Co 2p_3/2_ and Co 2p_1/2_, respectively, which are characteristic features of Co^2+^. The other two shake‐up peaks located at 785.1 and 802.5 eV are attributed to the satellite peaks (*), respectively. XPS data in **Figure** **2a** verify that Co ions in the as‐prepared Gly‐NCP are present in the form of Co^2+^. In Figure 2b, the peak located at 133.0 eV corresponds to the characteristic P 2p_3/2_ peak of P (V). The high‐resolution XPS spectra and curve fitting results of O 1s are shown in Figure 2c in which the predominant peak at 530.7 eV can be assigned to the PO_4_^3−^ ions, while the small peak at 532.4 eV is ascribed to crystal water. The appearance of a peak at 401.0 eV in Figure 2d is attributed to the N 1s, which is characteristic of NH_4_^+^ in the structure. Combined with the XRD results, XPS data further confirm that the as‐prepared sample was Gly‐NCP in nature.

### 2.2 OER Performance in Alkaline Media

**Figure 3:**
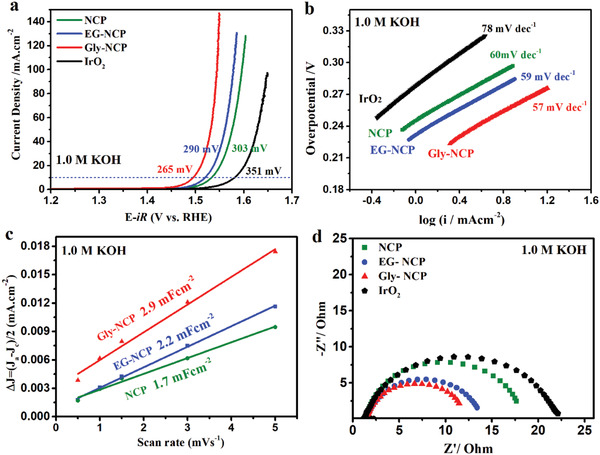
a) OER polarization curves and b) Tafel plots of NCP, EG‐NCP, Gly‐NCP, and IrO_2_ catalysts in 1.0 m KOH. c) Capacitive current densities of the different catalysts plotted against the scan rate and corresponding *C*_dl_ values estimated through linear fitting of the plots. d) Nyquist plots of different catalysts recorded at a given potential of 0.65 V (vs Hg/HgO) with the frequency range of 0.01–100 kHz.

The electrocatalytic activities of NCP catalysts toward the OER were investigated in 1.0 m KOH using the standard three‐electrode system. The current‐ and resistance‐ (*iR*) corrected polarization curves of NCP catalysts are shown in **Figure** **3a** which displays the geometric current density plotted against applied potential (vs reversible hydrogen electrode (RHE)). For comparison, the benchmark noble metal IrO_2_ catalyst was measured under the same condition. From linear sweep voltammetry (LSV) curves of catalysts in the alkaline media, it can be observed that the as‐prepared NCP, EG‐NCP, and Gly‐NCP catalysts show more superior catalytic activity toward the OER than that of IrO_2_. Meanwhile, the NCP nanosheets exhibit well‐differentiated OER activity according to their thickness. Particularly, Gly‐NCP displays excellent OER activity with lower onset potential and higher current density at the applied potential comparing with those of EG‐NCP and NCP catalysts. In OER performance evaluation, the overpotential required for delivering a current density of 10 mA cm^−2^ is usually compared. Strikingly, the Gly‐NCP requires an overpotential of only 265 mV to deliver 10 mA cm^−2^ (Figure 3a), which is lower than that of 290, 303, and 351 mV necessary for EG‐NCP, NCP, and IrO_2_ catalysts, respectively. This result indicates that the electrocatalytic OER activity of Gly‐NCP assembled with thinner nanosheets is significantly improved in comparison to those of EG‐NCP and NCP with thick nanosheets. Moreover, the electrocatalytic OER kinetics can be determined from Tafel plots and Figure 3b depicts the Tafel slopes of various catalysts. The Tafel slope of 57 mV dec^−1^ is achieved for Gly‐NCP, smaller than those of EG‐NCP (59 mV dec^−1^), NCP (60 mV dec^−1^), and reference IrO_2_ (78 mV dec^−1^), suggesting a more favorable OER kinetics and an excellent catalytic activity for Gly‐NCP catalyst. Additionally, the electrochemical active surface area (ECSA) is a significant parameter for catalysts in determining their electrocatalytic activity. In this study, the electrochemical double layer capacitance (*C*_dl_) (Figure S2, Supporting Information) was measured to evaluate the ECSA. Figure 3c shows that the *C*_dl_ for Gly‐NCP is 2.9 mF cm^−2^, higher than the *C*_dl_ demonstrated for EG‐NCP (2.2 mF cm^−2^) and NCP (1.7 mF cm^−2^). By adopting the specific capacitance of *C*_s_ = 0.040 mF cm^−2^ in 1.0 m KOH solution, the ECSA values of these samples are evaluated to be 42.5, 55.0, and 72.5 cm^2^ for NCP, EG‐NCP, and Gly‐NCP, respectively. Further, the ECSA normalized LSV curves of NCP, EG‐CNP, and Gly‐NCP toward the OER are considered.^[^ ^14^ ^]^ From Figure S3 (Supporting Information), it can be found that the Gly‐NCP shows better OER activity than those of EG‐NCP and NCP, which is consistent with the resulted LSV normalized via the electrode geometric area. The higher ECSA of Gly‐NCP consistently indicate that the Gly‐NCP possesses a larger electrochemical surface area, which enables more exposed active sites to take part in OER and hence achieves better electrochemical performance. Furthermore, to evaluate the electrode kinetics during the OER process, electrochemical impedance spectroscopy (EIS) was measured which provides the information on the interfacial reactions and behavior of catalysts. The EIS Nyquist results in Figure 3d show that the substantially reduced charge transfer resistance (*R*_ct_) was achieved for Gly‐NCP in comparison to the other catalysts. The R_ct_ of Gly‐NCP (1.53 Ω) is much smaller than that of EG‐NCP (1.56 Ω) and NCP (1.64 Ω), suggesting the rapid charge‐transfer kinetics between Gly‐NCP electrode and electrolyte, which contributes to the enhanced OER activity of the Gly‐NCP catalyst.

### 2.3 Electrochemical Performance of Alkaline Saline Water Oxidation

**Figure 4:**
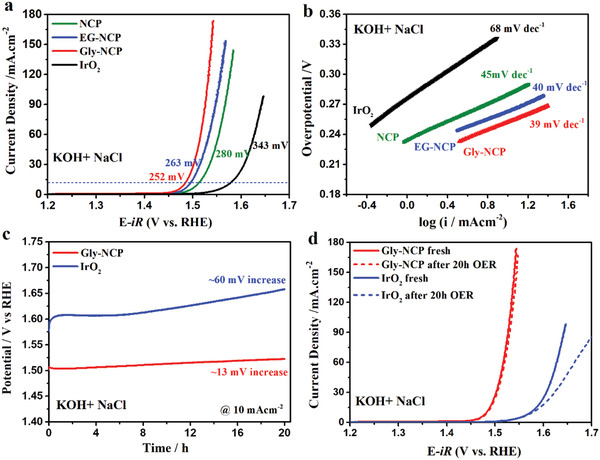
a) Saline water oxidation polarization curves and b) Tafel plots of different catalysts in alkaline simulated seawater of 1.0 m KOH+0.5 m NaCl. c) 20 h durability tests of Gly‐NCP and IrO_2_ catalysts at the constant current of 10 mA cm^−2^. d) Comparison of saline water oxidation activity of Gly‐NCP and IrO_2_ catalysts before and after durability test in 1.0 m KOH+0.5 m NaCl electrolyte.

Besides OER under freshwater condition, electrocatalytic seawater oxidation is also very attractive in terms of diversified future energy recourses, but this topic remains as a challenging task due to the poisoning of active sites and severe corrodibility of seawater against electrode, rendering the catalysts poorly active and very unstable. Therefore, it is of great significance to seek highly active and stable OER catalysts suitable for seawater oxidation. In this study, we further studied the OER activity of NCP, EG‐NCP, Gly‐NCP nanosheet catalysts in an alkaline simulated seawater electrolyte (1.0 m KOH+0.5 m NaCl). The LSV polarization curves and corresponding Tafel plots of carbon paper supported NCP and IrO_2_ electrodes are presented in **Figure** **4a**,b, respectively. Impressively, the lower overpotential (252 mV @ 10 mA cm^−2^) and lower Tafel slope (39 mV dec^−1^) demonstrate that the Gly‐NCP nanosheets have rapid OER catalytic kinetics and higher saline water oxidation activity than EG‐NCP (263 mV @ 10 mA cm^−2^), NCP (280 mV @ 10 mA cm^−2^), and it is also superior to the benchmark of IrO_2_ catalyst (343 mV @ 10 mA cm^−2^). The Gly‐NCP nanosheets with 30 nm thickness maintains its outstanding and even better OER activity in the NaCl‐containing electrolyte than in NaCl‐free 1.0 m KOH electrolyte. To clarify the better OER activity of Gly‐NCP catalyst in NaCl‐containing electrolyte, LSV curves of NCP catalysts before and after *i*R correction are analyzed in KOH and KOH+NaCl media, respectively. At the current density of 10 mA cm^−2^ before *i*R correction (Figure S4, Supporting Information), it is found that the Gly‐NCP electrode requires 15 mV lower overpotential and better OER activity in KOH+NaCl (272 mV) media than those in KOH (287 mV) electrolyte. After *i*R correction to eliminate the influence of electrolyte resistance, lower overpotential of 13 mV is illustrated by Gly‐NCP electrode in KOH+NaCl (252 mV) media than that in KOH (265 mV), which confirms the better OER activity of Gly‐NCP catalyst in NaCl‐containing electrolyte than in NaCl‐free 1.0 m KOH electrolyte. The mechanism for better OER activity in NaCl‐containing electrolyte is probably due to the diffusion of Cl^−^ and OH^−^ ions and intercalation/deintercalation into Gly‐NCP surface and interlayers,^[^ ^15^ ^]^ which play the role in decreasing the activation barrier and facilitating the intermediate adsorption/desorption, thereby promoting the O_2_ evolution and enhancing the OER activity. In addition, the actual overpotential (268 mV) applied on the Gly‐NCP electrode to achieve an OER current density of 100 mA cm^−2^ was well below the 480 mV overpotential required to trigger chloride oxidation to hypochlorite,^[^ ^7^, ^16^ ^]^ which suggests that chlorine ions in saline water show little effect on the OER performance of Gly‐NCP. Thesaline water oxidation performance of Gly‐NCP electrode outperforms most of the transition‐metal(oxy) hydroxide catalysts as well as many non‐noble metal catalysts (see comparison details in Table S1, Supporting Information). Furthermore, the overall saline water splitting performance was investigated using a two‐electrode configuration by pairing the OER catalyst of Gly‐NCP with another benchmark HER catalyst of Pt. As shown in Figure S5 (Supporting Information), to drive a current density of 10 mA cm^−2^ in 1.0 m KOH + 0.5 m NaCl, Gly‐NCP//Pt requires a small voltage of 1.712 V, which is 55 mV lower than that for the benchmarks of IrO_2_//Pt pair (1.767 V), further confirming the Gly‐NCP//Pt electrolyzer exhibits good activity for overall saline water splitting in the alkaline electrolyte.

Beyond the OER activity, stability is also an important factor of catalyst since it impacts the long‐term application of seawater electrolysis. As shown in Figure 4c, continuous chronoamperometric potential response is conducted at the current density of 10 mA cm^−2^. Strikingly, the potential required for Gly‐NCP to generate 10 mA cm^−2^ current density is merely 13 mV potential increases after 20 h of the continuous saline water oxidation. By contrast, the IrO_2_ catalyst caused 60 mV potential increases after 20 h of the stability test. Moreover, when comparing the LSV polarization curves of Gly‐NCP electrode before and after the stability test (Figure 4d), the polarization curves almost coincide with each other, indicating that the excellent saline water oxidation activity of Gly‐NCP nanosheet catalyst can be maintained, an evidence of its outstanding stability during alkaline saline water oxidation. Simultaneously, we examined the surface morphology and elemental distribution of the Gly‐NCP electrode after the stability test in 1.0 m KOH+0.5 m NaCl. SEM image and EDS maps (Figure S6, Supporting Information) prove that the nanosheet structure of Gly‐NCP catalyst is preserved after long‐term stability testing, attesting to the good structural stability of Gly‐NCP. Meanwhile, an ion chromatography (IC) test was performed to evaluate the side reaction of chlorine oxidation reaction to producing hypochlorite during the simulated seawater oxidation. As shown in Figure S7 (Supporting Information), the relative content of residual Cl^−^ shows insignificant change after the 20 h stability test, indicating the dominant OER without chlorine oxidation reaction during alkaline saline water oxidation. The good stability of Gly‐NCP can be mainly attributed to the following aspects: 1) the metal dissolution of transition metal phosphides is thermodynamically less favored under the experimental condition of this work, which endows the Gly‐NCP with enhanced corrosion resistance and chemical stability during the alkaline saline water oxidation; 2) There is no precipitate deposited on the active sites in our system which is a common reason for catalyst deactivation, thus alleviating the activity degradation; 3) Gly‐NCP catalyst exhibits high activity and low overpotential for the OER, which can sustain saline water oxidation at a potential well below that for chlorine evolution reaction. Combination of the above three features of Gly‐NCP guarantees its long‐term stability. Overall, the Gly‐NCP catalyst fabricated in this work has good activity and stability toward the OER in alkaline saline water and shows a great potential for rapid hydrogen production from seawater electrolysis without prepurification and desalination of seawater.

### 2.4 The Study of Active Sites and Reaction Mechanism

**Figure 5:**
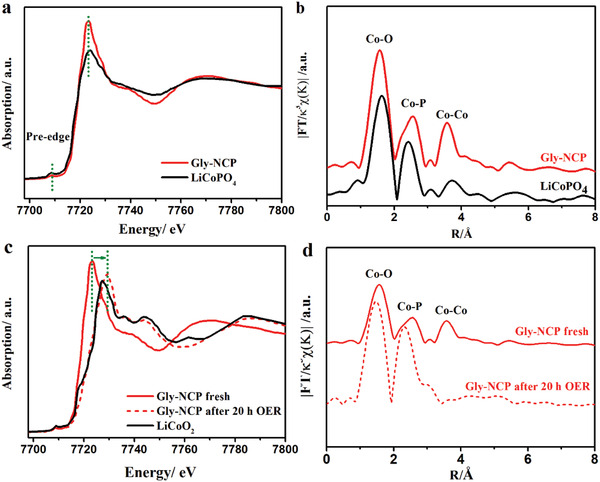
a) XANES spectra and b) FT‐EXAFS spectra of as‐prepared Gly‐NCP nanosheets and the reference of LiCoPO_4_. Comparison of c) the XANES spectra and d) FT‐EXAFS spectra of Gly‐NCP before and after 20 h saline water oxidation.

**Figure 6:**
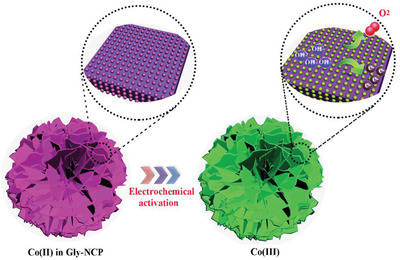
a) Schematic diagram for the electrochemical activation of Gly‐NCP for enhanced OER activity.

To gain further insights into the catalytic mechanism of the Gly‐NCP, X‐ray absorption near‐edge structure (XANES), extended X‐ray absorption fine structure (EXAFS), and the corresponding R‐space analysis were carried out on the Gly‐NCP before and after OER electrocatalysis. As shown in **Figure** **5a** and Figure S8 (Supporting Information), the Co K‐edge XANES spectra of the as‐prepared NCP, EG‐NCP, and Gly‐NCP exhibit characteristic edge absorptions at 7709 and 7719 eV, corresponding to the Co pre‐edge (Co 1s – O 2p hybrid orbitals) and Co 1s – Co 3d features, respectively. By comparing with the XANES of commercial LiCoPO_4_ powder sample as the reference, it can be inferred that Co ions in Gly‐NCP have similar chemical state as LiCoPO_4_ and are in the oxidation state of Co^2+^, which is consistent with the XPS result. The Fourier‐transformed (FT) EXAFS spectra of the reference LiCoPO_4_ exhibit several distinct peaks in R‐space. Briefly, the first FT peak observed at 1.57 Å is attributed to the local Co–O coordination, whereas another peak at 2.43 Å represents the related Co–P coordination as demonstrated in Figure 5b. The FT‐EXAFS of the as‐prepared Gly‐NCP exhibits the characteristic Co–O coordination (1.57 Å) and Co–P coordination (2.6 Å), indicative of the local atomic coordination of O atoms and P atoms around a central Co atom. As shown in Figure 5c, compared to the fresh Gly‐NCP catalyst, the tested Gly‐NCP after 20 h OER shows noticeable changes in the XANES spectra. The Co K‐edge white line peak demonstrates a clear edge jump shifting from 7719 eV to higher absorption energy of 7723 eV. This can be assigned to the formation of higher chemical states, similar to Co^3+^ in LiCoO_2_, indicating the oxidation activation of Co^2+^ to Co^3+^ during the OER electrocatalysis. From the cyclic voltammetry (CV) in Figure S9 (Supporting Information), an anodic activation peak near 1.2–1.3 V (vs RHE) corresponding to the Co^2+^→Co^3+^ can be observed for Gly‐NCP electrode in 1.0 m KOH+0.5 m NaCl. The current density is gradually increased over the initial CV cycles, indicating a possible surface activation during the OER, this is consistent with the result indicated by XAS. As reported in the literatures, the Co‐based anode would be transformed to Co (III) oxyhydroxide (Co–OOH) during the OER test, which actually serves as real active sites for the OER.^[^ ^12^, ^17^ ^]^ In order to unravel the coordination structure of the Gly‐NCP after 20 h OER, the local structure of Co atom in R space for the Gly‐NCP after OER is characterized by FT‐EXAFS and displayed in Figure 5d. After the OER measurement, the Co–P coordination shifted from 2.43 to 2.15 Å, while the Co–O coordination shifted from 1.6 to 1.48 Å, suggesting the increased valence state of Co ion to generate the shorter distance between Co–O and Co–P. During the OER processing, the Gly‐NCP is probably converted from crystalline to amorphous structures, which disrupts the long‐range ordered structure around Co and results the chemical states increase. The shifting of the Co K‐edge XANES confirms the conversion of Co^2+^ to Co^3+^ during the OER electrocatalysis by comparison with the Co^3+^ in LiCoO_2_. From the above results, it is believed that the excellent electrocatalytic activity of Gly‐NCP is attributed to the activation of Co (II) ions to Co (III) active sites, which is largely responsible for carrying out the OER activity (**Figure** **6**). Moreover, the advantageous features from the unique oriented stacking 2D nanosheets endow the Gly‐NCP with a large electrochemical active surface area and sufficient surface‐exposed Co (III) active sites, which favors the mass transport and promotes the OER kinetics, thereby leading to the excellent catalytic OER performance. Our results suggest that Gly‐NCP nanosheet is a promising catalyst candidate for both alkaline water and saline water oxidation, an achievement in fabricating high performance catalyst via economic and eco‐friendly route for seawater electrolysis.

## 3 Conclusions

In summary, the NCP nanosheets are prepared by a facile and lower energy consuming H_2_O‐mediated synthesis route at room temperature, which can be highly favorable for practical manufacturing. The Gly‐NCP assembled with optimal nanosheet thickness of 30 nm is obtained using H_2_O/glycerol mixed solvent. The unique oriented stacking 2D nanosheet structure endows Gly‐NCP with a large electrochemical active surface area, therefore, the high density of surface‐exposed active sites, which contributes to the enhanced OER performance in 1.0 m KOH. Meanwhile, Gly‐NCP catalyst exhibits outstanding electrocatalytic activity and stability toward the OER in challenging alkaline saline water media. XAS analysis conducted on the Gly‐NCP catalyst before and after alkaline saline water oxidation, demonstrated that the activation of Co (II) ions to Co (III) active sites is responsible for the excellent OER activity of Gly‐NCP nanosheet catalyst. The significance of this work lies in the development of Gly‐NCP nanosheets as a highly promising candidate catalyst for OER with great potential for practical seawater electrolysis for rapid hydrogen production without seawater prepurification and desalination.

## 4 Experimental Section

### Synthesis of Thickness‐Controlled NCP Nanosheets

NCP nanosheets were synthesized via chemical precipitation at room temperature. In a typical synthesis, 150 mg of ammonium dihydrogen phosphate (NH_4_H_2_PO_4_) and 360 *μ*L of 28 wt% ammonium hydroxides in H_2_O (NH_3_·H_2_O) were dissolved in 20 mL water, noted as solution A. To prepare solution B, 3 g ammonium chloride (NH_4_Cl) was first added and dissolved in 20 mL of water under magnetic stirring before 350 mg of cobalt chloride hexahydrate (CoCl_2_. 6H_2_O) was added. NH_4_Cl was used as the ammonium sources for synthesis of NCP, which contributes to the formation and precipitation of crystalline NCP nanosheets. Solutions A and B were mixed and stirred at room temperature for 12 h. The purple precipitates were formed, collected, and washed several times by DI water to remove the unreacted precursors. Finally, the obtained purple powder was dried at 50 °C in vacuum and named as NCP. To obtain thinner thickness of NCP nanosheets, the above procedure was repeated by replacing the solvent of 20 mL water with 10 mL of water + 10 mL of EG, or 10 mL of water + 10 mL of Gly under the same synthesis condition. The obtained samples were named as EG‐NCP and Gly‐NCP, respectively.

### Physical Characterization

The morphology and structure of NCP samples were characterized by using SEM (SU‐70), and TEM (JEM‐1230) equipped with EDS. XRD patterns were collected on a Bruker D8 Advanced diffractometer using Cu Ka radiation. XPS was conducted on a Thermo Scientific K‐Alpha spectrometer equipped with a monochromatic Al K*α* X‐ray source (1486.6 eV) operating at 100 W. The binding energies were calibrated relative to the C 1s peak energy at 284.8.0 eV. The XANES and EXAFS measurements at the Co K‐edge (7709 eV) were performed on the 061D superconducting wiggler hard X‐ray analysis beamline at the Canadian Light Source. The acquired XANES data were analyzed according to the standard procedures by using the Athena module. The EXAFS oscillation functions were obtained by subtracting the pre‐edge and postedge background from the overall absorption spectra, then normalized with respect to the edge‐jump step to unity. The extracted EXAFS data were analyzed by k^3^‐weighted *χ*(k) functions to obtain the magnitude plots in radial space.

### Electrode Preparation and Electrochemical Characterization

The catalyst inks were prepared by dispersing 5.0 mg of NCP, EG‐NCP, or Gly‐NCP and 5.0 mg carbon black (m_NCP_: m_carbon black_ = 1:1) in 5.0 mL of aqueous solution containing 1.0 mL of isopropyl alcohol and 50 µL of Nafion (5.0 wt%). Then 30 min sonication was conducted to ensure uniform dispersion and wetting of the catalyst powder (1.0 mg mL^−1^). 1.0 mL of the catalyst ink was loaded onto the surface of carbon paper (1.0 × 1.0 cm^2^) via spray‐coating and allowed to dry at room temperature. The NCP catalyst loading on the carbon paper supported electrode was controlled to be 1.0 mg cm^−2^. For comparison purpose, Ir‐based catalyst was taken as the benchmark catalyst for OER, and the carbon paper (1.0 × 1.0 cm^2^) supported commercial IrO_2_/C (20 wt%) catalyst with loading of 1.0 mg cm^−2^ was prepared with the same spray‐coating method.

All electrochemical measurements were conducted with CHI760e potentiostat with a three‐electrode system. The carbon paper supported NCP catalyst, Pt wire, and saturated Hg/HgO were used as the working electrode, counter electrode, and reference electrode, respectively. The catalytic activity for the OER was measured in aqueous 1.0 m KOH (pH 13.8) and 1.0 m KOH+0.5 m NaCl (mimic alkaline seawater) ^[^ ^7^, ^8^ ^]^ electrolyte at room temperature, respectively. Polarization curves of the working electrode were obtained by performing the LSV measurements at a scan rate of 10 mV s^−1^. All the potentials were converted to the RHE potential scale through calibration: E(RHE) = E(Hg/HgO) + 0.0591 *pH + 0.098, where E(Hg/HgO) is the measured potential (vs Hg/HgO). Measured potentials were corrected with *iR* compensation: E = E(RHE)‐ *iR*, where *i* is the measured current density (mA cm^−2^), and *R* is the solution resistance. The Tafel slope was calculated according to the equation *η*  =  b*(log *i*) + *a*, where *η* is the overpotential (V), and *b* is the Tafel slope (mV dec^−1^). EIS was undertaken at 0.65 V (vs Hg/HgO) with AC amplitude of 10 mV and a frequency range of 0.01–100 kHz. Long‐term stability tests were carried out at the constant current density of 10 mA cm^−2^ at room temperature.

## Conflict of Interest

The authors declare no conflict of interest.

## Supporting information

Supporting Information

## Data Availability

The data that support the findings of this study are available from the corresponding author upon reasonable request.
