# Supplementary material for: High‐Performance Ammonium Cobalt Phosphate Nanosheet Electrocatalyst for Alkaline Saline Water Oxidation
Source: Adv Sci (Weinh). 2021 May 16;8(14):2100498. doi: 10.1002/advs.202100498 (PMC8292903; doi:10.1002/advs.202100498)
Supplement: Supplementary file 1 — Supporting Information [file ADVS-8-2100498-s001.pdf]

© 2021 Wiley-VCH GmbH

ADVANCED SCIENCE
Open Access

Supporting Information

for *Adv. Sci.*, DOI: 10.1002/advs.202100498

High-performance Ammonium Cobalt Phosphate Nanosheet Electrocatalyst for Alkaline Saline Water Oxidation

Zhongxin Song [a], Kaixi (Cathy) Wang [bc], Qian Sun [b], Lei Zhang [b], Junjie Li [b], Dingjiu Li [a], Pok-Wai Sze [a], Yue Liang [a], Xueliang Sun [b], Xian-Zhu Fu [a], Jing-Li Luo [a,]*

Supporting Information

High-performance Ammonium Cobalt Phosphate Nanosheet Electrocatalyst for Alkaline Saline Water Oxidation

Zhongxin Song [a], Kaixi (Cathy) Wang [bc], Qian Sun [b], Lei Zhang [b], Junjie Li [b], Dingjiu Li [a], Pok-Wai Sze [a], Yue Liang [a], Xueliang Sun [b], Xian-Zhu Fu [a], Jing-Li Luo [a,]*

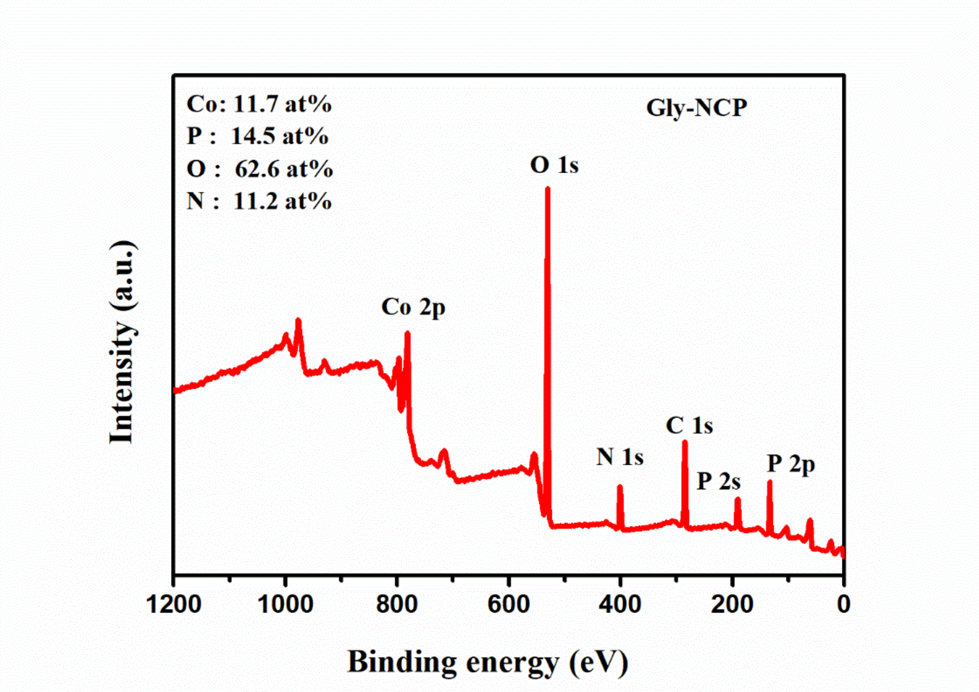

Figure S1. The XPS spectrum and element composition of as-prepared Gly-NCP.

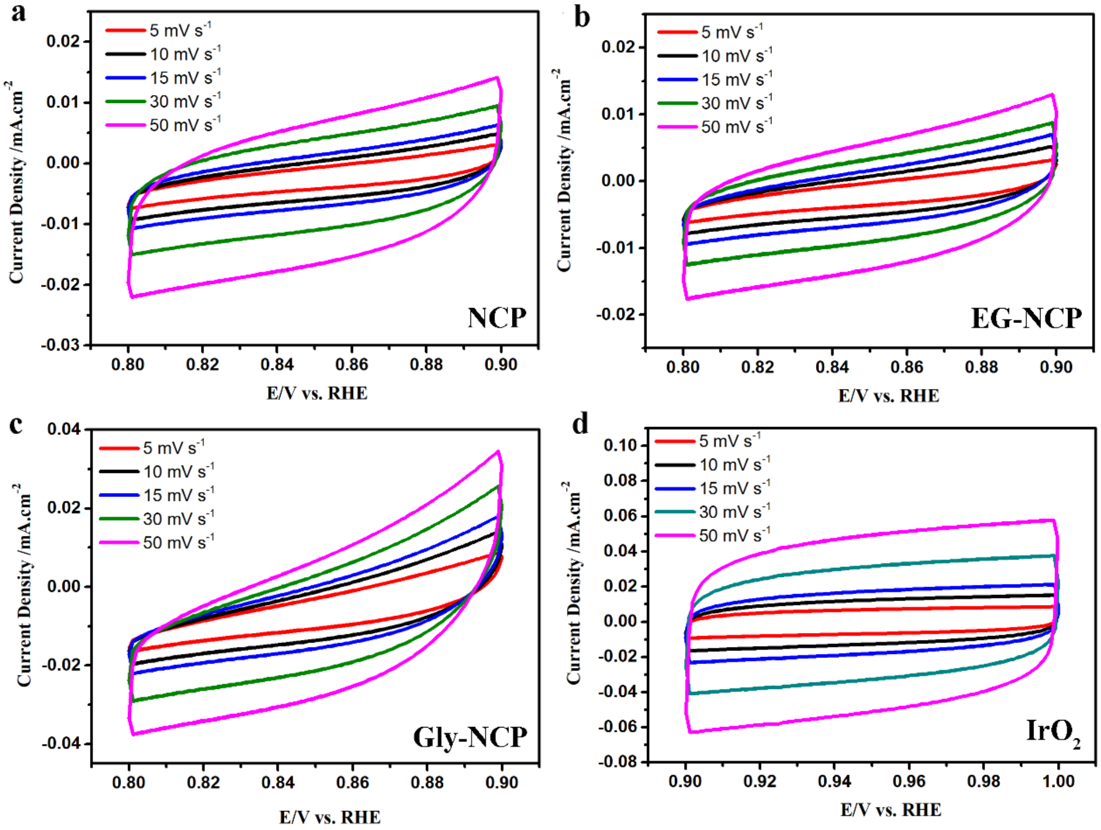

Figure S2. The CV curves at different scan rates for NCP, EG-NCP, Gly-NCP nanosheets and the reference of IrO_2.

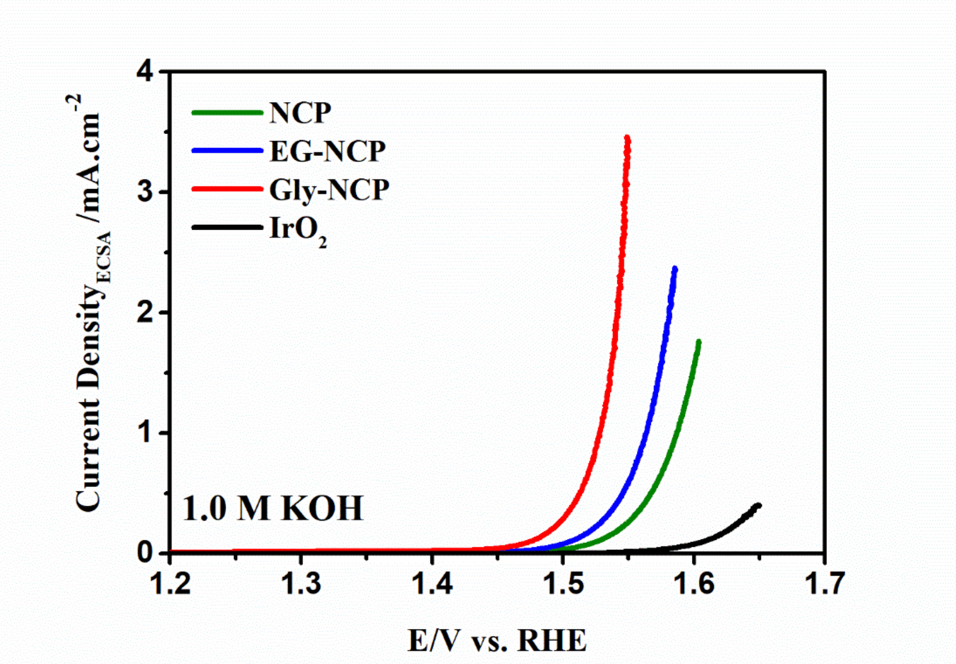

Figure S3. The ECSA normalized LSV curves for NCP, EG-NCP, Gly-NCP nanosheets (scan rate: 10 mVs^{-1})

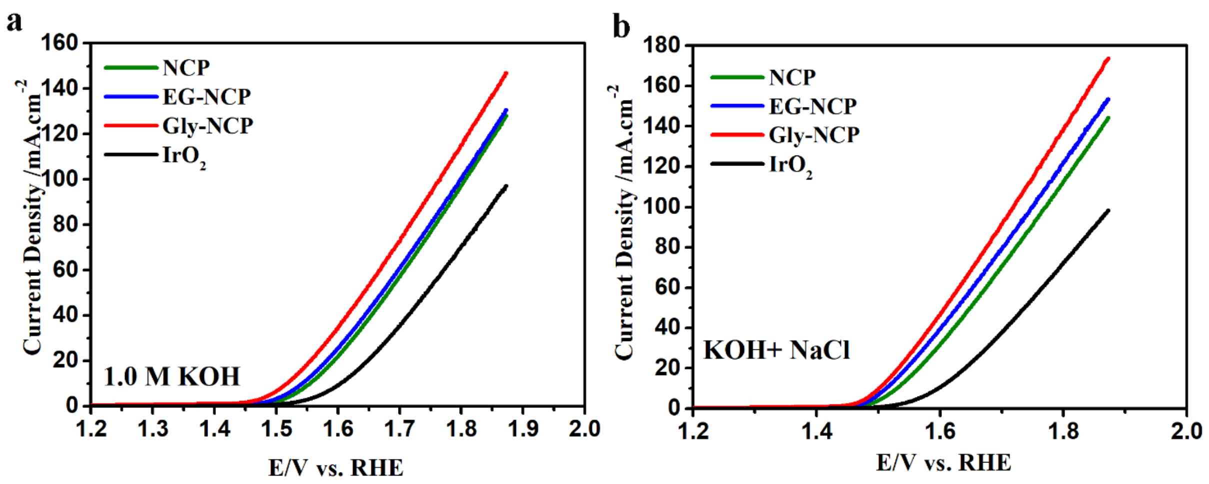

Figure S4. The LSV curves for NCP, EG-NCP, Gly-NCP nanosheets in (a) 1.0 M KOH and (b) 1.0 M KOH+0.5 M NaCl before *j*R correction (scan rate: 10 mV s^{-1}).

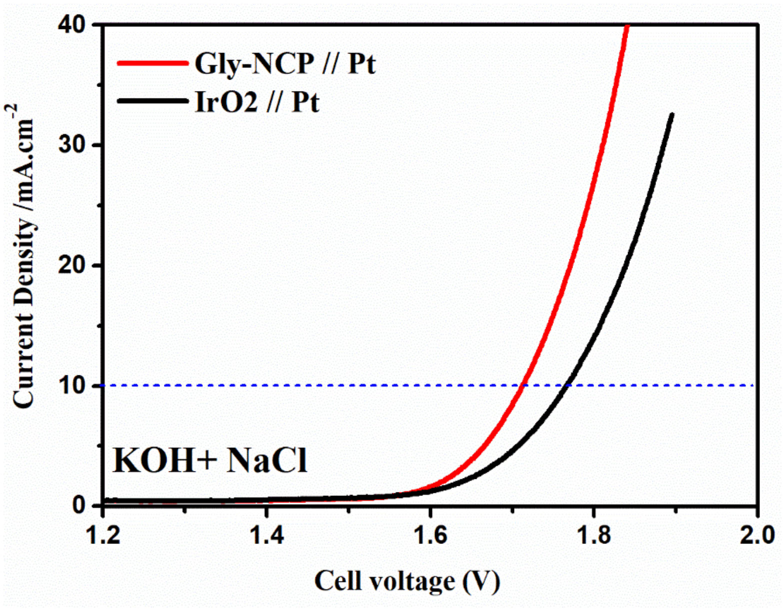

Figure S5. The LSV curve for overall alkaline saline water electrolysis of Gly-NCP//Pt and IrO_2//Pt under two electrode system (scan rate: 10 mVs^{-1}).

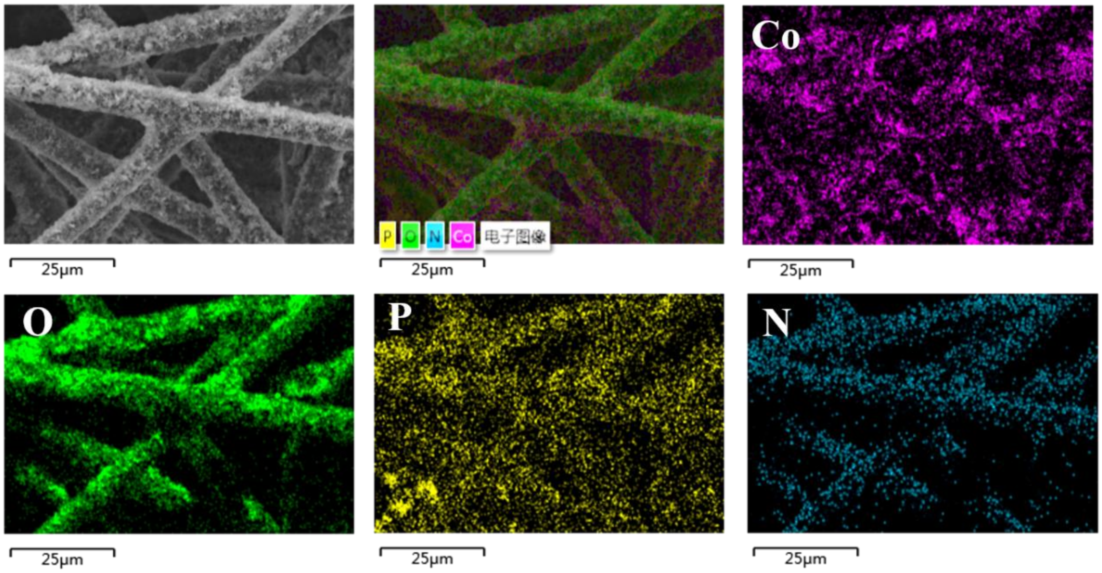

Figure S6. The SEM image and EDS maps of Gly-NCP electrode after 20 hours of stability test in 1.0 M KOH+0.5 M NaCl.

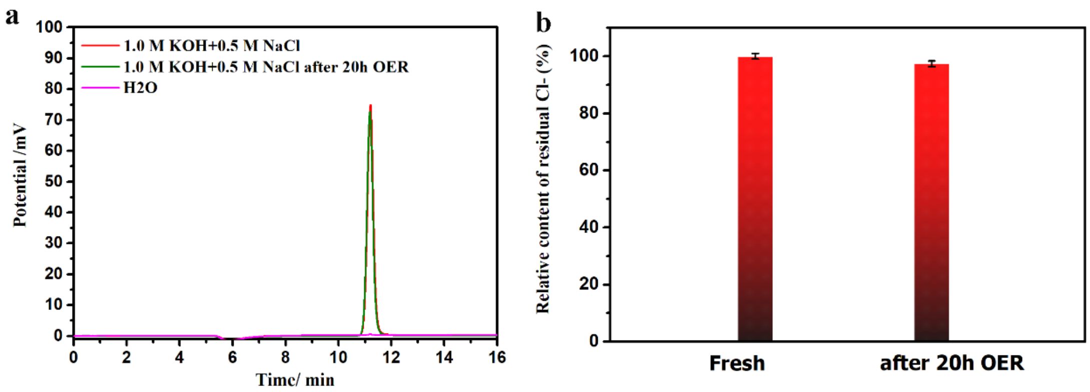

Figure S7. The Ion-Chromatography traces of diluted electrolyte (a) and relative content of residual Cl^- in KOH+NaCl electrolyte (b) before and after 20 h OER test catalyzed by Gly-NCP at 10 mA cm^{-2}.

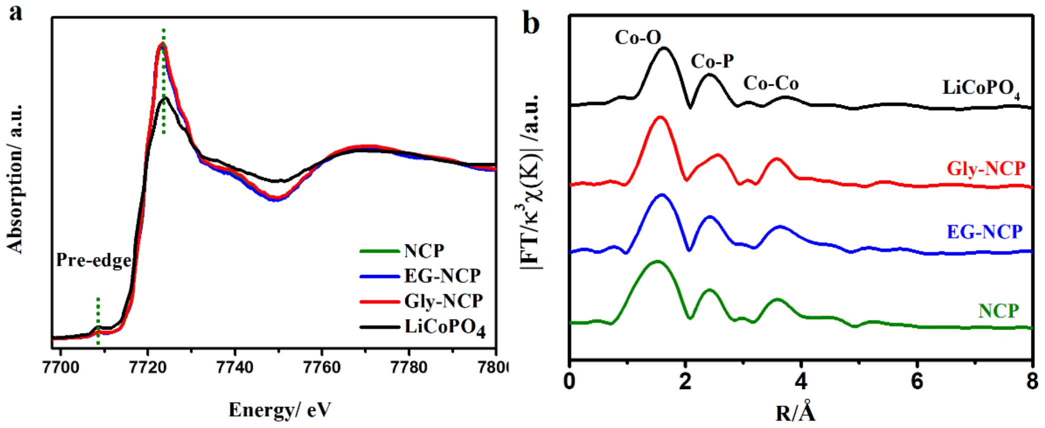

Figure S8. (a) XANES spectra and (b) FT-EXAFS spectra of as-prepared NCP, EG-NCP, Gly-NCP nanosheets and the reference of $LiCoPO_4$.

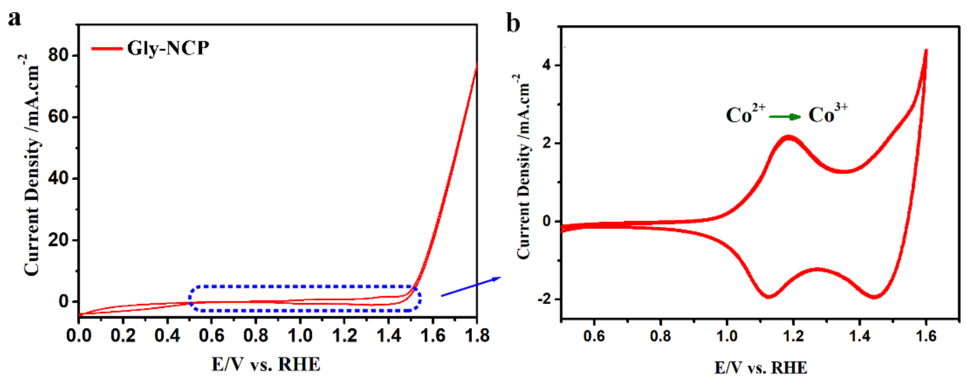

Figure S9. The CV curve of Gly-NCP electrode in 1.0 M KOH+0.5 M NaCl (scan rate: 50 mVs^{-1}).

Table S1. Comparison of electrocatalytic activity towards seawater oxidation for recently reported electrocatalysts

Electrocatalysts	Electrolyte	Overpotential	Tafel slope $mVdec^{-1}$	Stability	Reference
3D core-shell NiMoN@NiFeN	1.0 M KOH + 0.5 M NaCl	286 mV@ 100 mA cm^{-2}	-	Current increase <10% after 100 h	[1]
$Na_2Co_{1-x}Fe_xP_2O_7$	0.5 M NaCl + 0.1 M KOH	285 mV @ 100 mA cm^{-2}	56	40 mV increase after 100 h	[2]
Ni_2P-Fe_2P	1 M KOH seawater	305 mV @ 100 mA cm^{-2}	-	36 h stable at 100 mA cm^{-2}	[3]
NiFe/NiS_x-Ni foam	1 M KOH+ 0.5 M NaCl	380 mV @ 1500 mA cm^{-2}	-	500 h stable at 400-1000 mA cm^{-2}	[4]
S-(Ni,Fe)OOH	1 M KOH+ 0.5 M NaCl	278 mV @ 100 mA cm^{-2}	48.9	100 h stable at 100 mA cm^{-2}	[5]
NiFe-LDH	0.1 M KOH+0.5m NaCl	359 mV @ 10 mA cm^{-2}	50	480 mV increase after 2h operation	[6]
NiCo-DEA	seawater	Onset potential of 1.31 V	51	Keep 96% current after 8h at 1.5 V	[7]
NiNS	Overall seawater splitting	48.3 mA cm^{-2} at 1.8 V	112	Stable during 12 h operation	[8]
Gly-NCP	1.0 M KOH + 0.5 M NaCl	268 mV @ 100 mA cm^{-2}	39	13 mV increase after 20h operation	This work

References

[1] L. Yu, Q. Zhu, S. W. Song, B. McElhenny, D. Z. Wang, C. Z. Wu, Z. J. Qin, J. M. Bao, Y. Yu, S. Chen, Z. F. Ren, *Nat. Commun.* **2019**, 10, 10.

[2] H. J. Song, H. Yoon, B. Ju, D. Y. Lee, D. W. Kim, *Acs Catalysis* **2020**, 10, 702.

[3] L. Wu, L. Yu, F. Zhang, B. McElhenny, D. Luo, A. Karim, S. Chen, Z. Ren, *Adv. Funct. Mater.* **2021**, 31, 2006484.

[4] Y. Kuang, M. J. Kenney, Y. T. Meng, W. H. Hung, Y. J. Liu, J. E. Huang, R. Prasanna, P. S. Li, Y. P. Li, L. Wang, M. C. Lin, M. D. McGehee, X. M. Sun, H. J. Dai, *Proc. Natl. Acad. Sci. U. S. A.* **2019**, 116, 6624.

[5] L. Yu, L. Wu, B. McElhenny, S. Song, D. Luo, F. Zhang, Y. Yu, S. Chen, Z. Ren, *Energy Environ. Sci.* **2020**, 13, 3439.

[6] F. Dionigi, T. Reier, Z. Pawolek, M. Gliech, P. Strasser, *ChemSusChem* **2016**, 9, 962.

[7] J. J. Zheng, *Electrochim. Acta* **2017**, 247, 381.

[8] Y. Q. Zhao, B. Jin, A. Vasileff, Y. Jiao, S. Z. Qiao, *Journal of Materials Chemistry A* **2019**, 7, 8117.
